# Recent Advances in Multitone Microwave Frequency Measurement

**DOI:** 10.3390/s25123611

**Published:** 2025-06-09

**Authors:** Md Abu Zobair, Behzad Boroomandisorkhabi, Mina Esmaeelpour

**Affiliations:** Department of Electrical and Computer Engineering, Missouri University of Science and Technology, Rolla, MO 65409, USA

**Keywords:** microwave photonics, instantaneous frequency measurement, optical processing

## Abstract

This review explores various advanced photonic-assisted techniques for microwave frequency measurement, highlighting their distinct advantages and challenges in detecting multi-tone and broadband microwave frequency signals. Different optical processing techniques for instantaneous frequency measurement, including frequency-to-time mapping techniques, are discussed in detail. The application of multicore and few-mode fibers, artificial intelligence-enhanced, and complex modulation techniques are also discussed. These recent advances collectively push the boundaries of microwave frequency measurement, offering robust and scalable solutions for various applications.

## 1. Introduction

Over the years, methods for microwave frequency measurement (MFM) have evolved significantly, transitioning from traditional mechanical and electronic approaches limited by their precision and range to more sophisticated photonics techniques [1,2,3,4]. Photonic-based instantaneous frequency measurement (IFM) systems represent an innovative approach, utilizing various optical effects and devices to achieve wideband and high-precision single-tone, multi-tone, and broadband frequency measurements. These systems can attain MHz-range accuracy across tens of GHz frequency bandwidth, demonstrating exceptional stability and noise performance. Integrating complex microwave photonic circuits into compact photonic chips promises further advancements in this field, enhancing performance and applicability [5,6,7]. Machine learning (ML) techniques have also recently been employed to enhance the precision of such systems, significantly reducing the required experimental data and improving frequency measurement accuracy. The ML integration showcases the potential for intelligent systems to adapt and optimize measurement processes, leading to more accurate and reliable outcomes [8,9]. This review highlights the recent advances in photonics-based IFM techniques capable of detecting multi-tone signals, emphasizing their principles, advantages, and challenges in advancing precision and stability in frequency measurement technology. By exploring these advancements, we gain a deeper understanding of the critical role that multi-tone frequency measurement plays in various scientific and engineering domains, paving the way for future innovations.

## 2. Microwave Frequency Measurement Techniques

**Figure 1:**
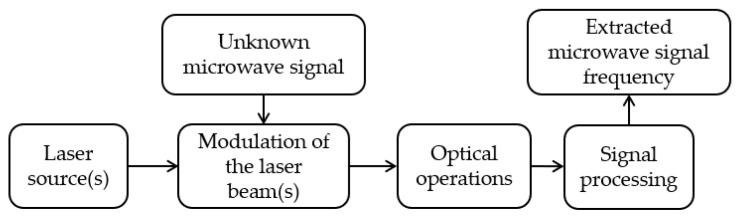
Functional block diagram of a general photonics-based MFM system.

A photonics-based MFM or IFM system comprises four principal blocks (see Figure 1): the optical source, the electrical-to-optical conversion, optical processing, and optical-to-electrical conversion followed by signal processing. The light source supplies the optical carriers, which may be single-wavelength or broadband continuous-wave (CW) lasers, mode-locked lasers of broadband spectrum, or optical frequency comb (OFC) laser sources. The electrical-to-optical conversion transforms the microwave frequency signal into the optical domain through amplitude, phase, or polarization modulations. The optical processing constitutes the core of the frequency measurement system, where the primary signal processing operations occur within the optical domain and may involve various optical operations such as interference and filtering. Following optical processing, the outputs are formatted for detection and frequency interpretation using digital signal processing (DSP).

Optical processing techniques for MFM can generally be categorized as techniques that map frequency to power, time, or space. The frequency-to-space mapping (FTSM) technique, also known as channelization, divides the frequency components of the microwave signal into various spatially limited channels [10,11,12,13,14]. In general, channelization is a useful technique for accurately determining the frequency information by determining the channels in which the received multiple signals are placed. The frequency-to-time mapping (FTTM) technique directly maps the frequency of the unknown signals to a distinct set of relative time delays [15,16]. In the frequency-to-power mapping (FTPM) technique, the carrier frequency of the microwave signal is determined from the unique mapping between the output amplitude and the microwave frequency [17,18]. Oftentimes, this relation is estimated through a microwave power function defined as the amplitude comparison factor (ACF). Additionally, frequency-to-phase and frequency-to-phase-slope mappings have also been demonstrated to measure broadband frequency signals and address the shortcomings of the FTPM techniques by avoiding frequency measurement ambiguity [19,20,21,22,23]. Among these mapping techniques, FTPM has been extensively studied, and many different optical processing techniques have been developed to improve its performance, including measurable frequency range, accuracy, and resolution. We refer the readers to the cited review papers for a more in-depth study of these techniques [1,2,3,4,24,25,26,27].

The FTPM techniques are mainly capable of detecting single-tone frequency signals, while the FTTM, FTSM, and hybrid techniques that combine FTTM and FTSM with FTPM techniques can detect multi-tone and broadband frequency signals. The FTTM techniques excel in providing high measurement resolution and wide-frequency-range capabilities and have demonstrated enhanced performance in terms of speed and accuracy over traditional electronic methods. Another crucial aspect is that FTTM benefits from the robustness inherent in photonics-based systems, which are less susceptible to electromagnetic interference compared to conventional electronic systems, thereby facilitating effective operation in harsh environments [28]. This resilience reinforces the potential for FTTM to be integrated into applications such as radar and telecommunications, where frequency measurements are critical. Notably, FTTM implementations also benefit from reductions in hardware complexity and size, which are essential for applications within space-constrained environments, such as airborne systems [29]. These characteristics contribute to the design of compact photonic systems that accomplish measurement tasks with minimal structural overhead while maintaining substantial measurement fidelity [30]. On the other hand, FTSM techniques are advantageous for their real-time capabilities and efficiency in handling multi-tone broadband signals. As noted by Zhu et al. [31], FTSM employs techniques such as Fabry–Pérot filters and fiber Bragg gratings, allowing for the simultaneous monitoring of multiple frequencies. This capacity for multi-frequency measurement significantly enhances functionality, making FTSM ideally suited for modern applications requiring high fidelity in measuring complex signals. Furthermore, FTSM’s ability to map signal frequencies into spatial dimensions provides intuitive insights into the signal’s characteristics, offering broader situational awareness in monitoring scenarios [32].

**Figure 2:**
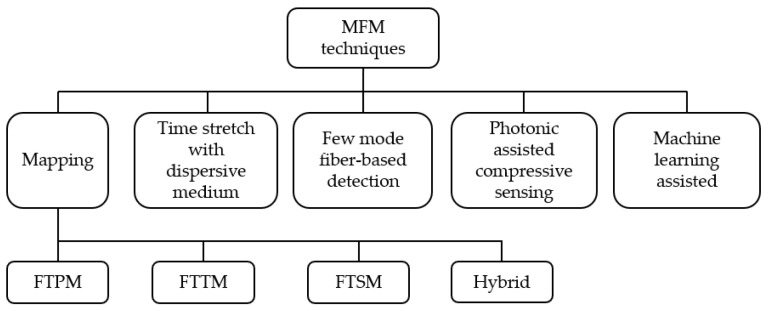
Tree diagram of different MFM techniques.

In the most recent advancements, MFM has been demonstrated using time-stretch with dispersive elements [33,34], hybrid techniques combining the advantages of FTTM and FTSM techniques [35,36,37], and artificial intelligence to improve measurement resolution, accuracy, and bandwidth [38]. Additionally, new fiber types, such as few-mode fibers (FMFs), have been utilized for MFM [39], with the potential for broadband and multi-tone frequency detection, and on-chip solutions for miniaturization have been improved [40] to reduce the cost of broader dissemination. Recently, the photonic compressive sensing of microwave signals also emerged as an effective technique for MFM [41,42]. Below, we review the recent advancements in photonic-assisted MFM techniques for multi-tone and broadband signals to highlight their strengths and challenges, providing a comprehensive understanding of their practical applications and limitations. The advancements in FTTM based on filtering and scanning techniques, FTSM, and hybrid techniques combining FTTM with FTSM and FTPM will be discussed. Figure 2 lists the techniques that will be discussed in the following.

### 2.1. FTTM Based on Stimulated Brillouin Scattering

**Figure 3:**
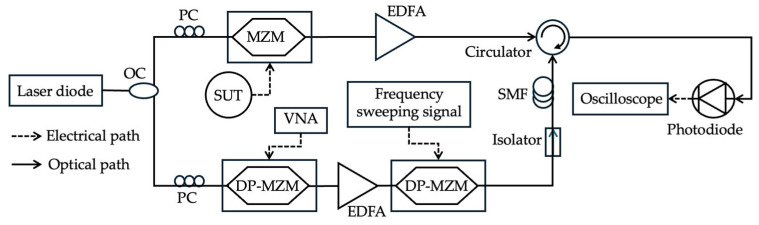
Experimental setup of the proposed multiple microwave frequency measurement system (adapted from [15]). OC: optical coupler; PC: polarization controller; EDFA: erbium-doped fiber amplifier; VNA: vector network analyzer.

The stimulated Brillouin scattering (SBS) gain spectrum can create narrowband filters for the FTTM techniques. This effect results in a frequency-shifted signal whose frequency is downshifted by a few tens of GHz from the pump laser frequency. The SBS gain spectrum is usually a result of the interaction between a pump wave modulated by the unknown microwave signal and a counter-propagating frequency sweeping optical probe wave. The SBS gain spectrum acts as a sweeping narrowband microwave photonic filter (MPF). Liu et al. [15] utilized SBS to perform FTTM operation by measuring the Brillouin gain spectrum using a frequency-sweeping optical probe wave. As shown in Figure 3, the output of a laser diode is divided into two paths referred to as pump and probe. In the pump path, the laser intensity is modulated with an unknown microwave frequency signal to generate a carrier-suppressed double-sideband (CS-DSB) signal. In the probe path, the laser beam goes through two cascaded dual-parallel Mach–Zehnder modulators (DP-MZMs). The first MZM is modulated by the Brillouin frequency shift in the dispersive fiber used in the setup and the second MZM is modulated by a frequency sweeping signal operating at carrier-suppressed single-sideband (CS-SSB) regime. The pump and probe signals go through a length of single-mode fiber (SMF) in opposite directions. The probe is amplified when the sweeping frequency matches the unknown signal frequency gain peak generated by the unknown signal in the pump path. The unknown frequency can be determined by introducing a known reference signal based on the time difference between the pulses of the unknown and reference signals. A measurement range of 6–18 GHz with a maximum error of ±1 MHz and resolution of 20 MHz was achieved. Jiang et al. [43] used a centimeter-scale chalcogenide glass waveguide to generate the SBS-based MPF and achieved an estimated range of up to 38 GHz with an error lower than 1 MHz. Long et al. [44] experimentally demonstrated a bandwidth of 27.5 GHz and a frequency error within 20 MHz.

### 2.2. FTTM with Phase Modulation and Microwave Photonic Filter

**Figure 4:**
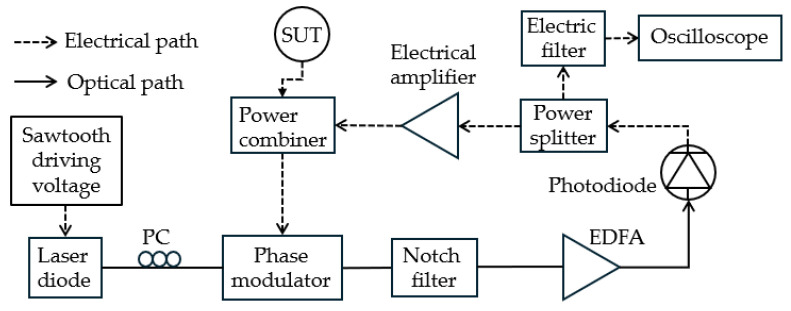
The schematic diagram of the frequency measurement system based on FDML-OEO (adapted from [45]).

Another FTTM technique capable of multi-tone MFM was developed by phase modulation and filtering. Hao et al. [45] demonstrated an FTTM technique using a Fourier domain mode-locked optoelectronic oscillator (FDML-OEO), as shown in Figure 4. The FDML-OEO is based on a CW laser diode driven by a sawtooth signal, resulting in fast-frequency scanning of the laser. The resulting pulses are then phase-modulated by the unknown microwave frequency signal followed by a phase-shifted fiber Bragg grating (FBG) utilized as the notch filter, converting the phase modulation to intensity modulation. A photodiode converts the optical signal to an electrical signal, which is then split. An oscilloscope detects part of the signal after passing an electric filter, and part of it is combined with the unknown microwave signal, resulting in an interference signal fed into the phase modulator. This generates frequency-scanning microwave signals followed by an MPF. The electrical filter selects a portion of the beat-note or sum-note between the signal under test (SUT) and the frequency scanning signal of the FDML-OEO. The frequency of the unknown microwave signals is thus mapped to the time difference between the output pulses. A 15 GHz measurement range with a 60 MHz maximum error was achieved.

**Figure 5:**
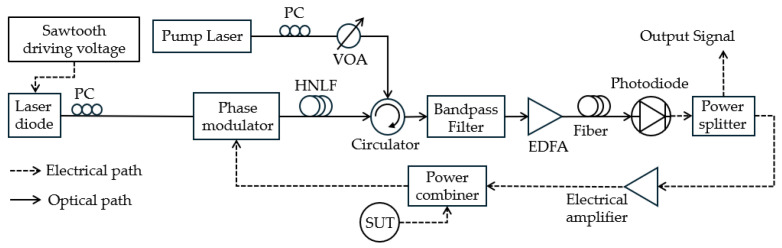
The schematic diagram of the frequency measurement system based on SBS (adapted from [46]). PC: polarization controller; EDFA: erbium-doped fiber amplifier.

Using a highly nonlinear fiber (HNLF) and its SBS gain profile, Hao et al. [46] achieved a 16 GHz measurement range and 0.07 GHz measurement error (Figure 5). The frequency measurement range of this technique can be tuned by adjusting the DC bias or amplitude of the driving signal of the SBS-based MPF. The key advantages are that it eliminates the need for an external electrical filter, simplifies the system, and enables multi-tone frequency measurement with broad bandwidth, high-resolution, and tunable measurement range. Table 1 lists some of the most recent publications on FTTM techniques based on SBS and FDML OEO for multi-tone MFM, their range, resolution, and accuracy or error. Table 1 compares the published results of different SBS and OEO-based MFM techniques.

### 2.3. FTTM with Ring Resonator Filtering

**Figure 6:**
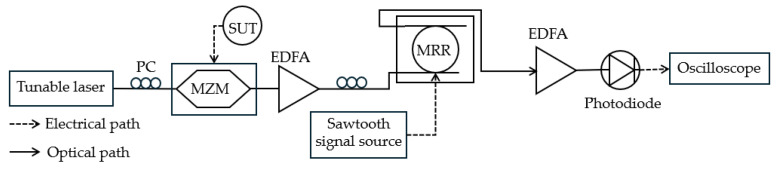
The schematic diagram of the frequency measurement system based on the MRR filter (adapted from [52]). PC: polarization controller; EDFA: erbium-doped fiber amplifier.

A ring resonator can be utilized as an MPF for MFM. Zhou et al. [52] used a swept frequency high-quality-factor silicon micro-ring resonator (MRR) as a periodic narrowband scanning filter driven by a sawtooth voltage signal (Figure 6). The FTTM was achieved by mapping the unknown frequency to the time interval between pulse appearances when scanning the modulated signal. In this technique, the CW tunable laser (*f*_0_) is intensity modulated by the unknown microwave frequency signal (*f*_1_), generating sidebands of *f*_0_ + *f*_1_ and *f*_0_ − *f*_1_. The modulated signal passes through the drop port of the MRR, the resonant peak of which acts as a narrowband filter. A sawtooth voltage signal is applied to the MRR’s electrodes, causing its resonant peak to periodically blue shift, effectively creating a periodic scanning filter. As the filter scans across the frequencies of the modulated signal (carrier and sidebands), each frequency component is selectively passed through the filter at different times, resulting in intensity peaks detected by a low-speed photodetector and observed on an oscilloscope. The time intervals between the appearances of these intensity peaks correspond to the frequency differences between the carrier and the sidebands, thus mapping frequency to time. An FTTM function is established to derive the unknown frequency (*f*_1_) from the measured time intervals. This function depends on the voltage-to-time relationship of the sawtooth signal and the frequency-to-voltage characteristic of the MRR, which needs to be measured and fitted in advance. For multi-frequency measurement, each unknown frequency component in the input signal will also be mapped to a corresponding time interval, allowing for the simultaneous detection of multiple frequencies. However, the resolution for distinguishing multiple frequencies is limited by the 3 dB bandwidth of the MRR filter. The system achieves a 25 GHz measurement range (limited by the bandwidth of the microwave signal source used in the experiments) with a ±510 MHz error and a 5 GHz multi-frequency resolution (limited by the 3 dB bandwidth of the MRR filter), offering advantages in simplicity, cost, and the potential for integration. Based on the suppression band of the MRR, the system can potentially achieve MFM up to 40 GHz.

**Figure 7:**
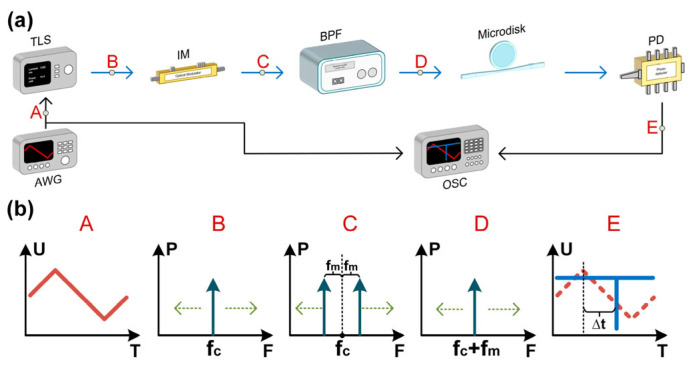
(**a**) Schematic diagram of the frequency measurement principle [53]. AWG: arbitrary waveform generator; TLS: tunable laser source; IM: intensity modulator; BPF: bandpass filter; PD: photodetector; OSC: oscilloscope. (**b**) The corresponding waveform or spectrum diagram at points A, B, C, D, and E marked in the schematic diagram.

Zhao et al. [53] used an ultrahigh-quality-factor magnesium fluoride micro-disk resonator (MDR) to perform FTTM operation (Figure 7). A frequency-swept tunable laser driven by a triangular waveform is intensity-modulated in the carrier-suppressed operational regime. A bandpass filter selects the upper modulation sideband and feeds the signal into an MDR. When the chosen sideband matches the optical frequency of the resonator, a dip appears on the photodetector trace. The unknown microwave frequency is linearly related to the time difference between the dip and the falling edge of the triangular wave. A 3 GHz measurement bandwidth from 14.25 to 17.25 GHz with a less than 10 MHz error was achieved for single-frequency measurement. The range can potentially be extended by optimizing coupling to utilize the MDR’s free spectral range (FSR, around 10 GHz) or by recalibrating for different radio frequency (RF) ranges. The root mean square (RMS) error is calculated to be 4.8033 MHz, attributed to the MDR’s exceptional quality factor (exceeding 10^8^). The measurement speed is determined by the sweep frequency of the tunable laser source, which is set to 32 Hz, resulting in a period of 31.25 ms.

**Figure 8:**
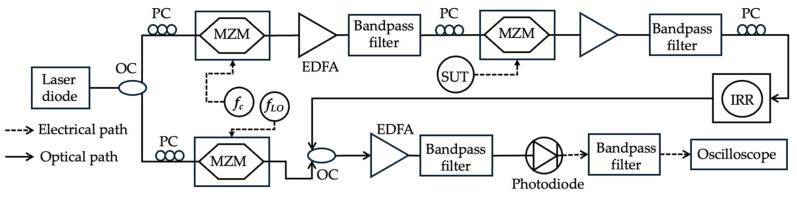
The schematic diagram of the frequency measurement system based on IRR (adapted from [54]). OC: optical coupler; PC: polarization controller; EDFA: erbium-doped fiber amplifier.

Singh et al. [54] demonstrated a measurement accuracy of ±0.4 MHz and a resolution of less than 1 MHz (defined by the linewidth of the electrical bandpass filter used) over a frequency range of 10 GHz using an integrated ring resonator (IRR) combined with heterodyne detection (Figure 8). A CW laser is divided into two arms of a Mach–Zehnder interferometer. In the upper arm, the carrier wave (*f_c_*) is modulated with a sweep frequency using an MZM operating at carrier suppression. The output is then modulated again with the SUT in a second MZM. A part of the upper sideband of this signal is filtered using a narrow linewidth-integrated ring resonator on a silicon nitride platform. The unknown signal’s spectrum is effectively time-swept through the narrow linewidth of the ring resonator. In the interferometer’s lower arm, the carrier is modulated by another MZM to generate a local oscillator (LO) wave (*f_LO_*) for heterodyne signal detection. The *f_LO_* has a constant frequency shift (Δ*f*) from the center of the ring resonator (*f_LO_ = (f_resonance_* − *f_c_) +* Δ*f*). The filtered part of the SUT and the LO are combined and converted to the electrical domain using a photodiode. This process results in a baseband spectrum and an up-converted version at frequency Δ*f*. The up-converted spectrum at Δ*f* is then filtered by a narrow linewidth electrical band-pass filter (BPF) with a central frequency equal to Δ*f*. As the whole spectrum of the SUT is swept through the ring resonance, the output current after the BPF, measured over sweeping time, represents the spectrum of the SUT. The sweep rate (*k*) is crucial for mapping the frequencies in the original signal to the measured time-domain trace. It is calculated as (*f_stop_* − *f_start_*)/Δ*t*, where Δ*t* is the sweep duration. An estimation of the unknown frequencies is obtained by multiplying the temporal difference in each signal component by the sweep rate. The *f_stop_* and *f_start_* of the sweep are known based on the laser frequency and IRR resonance. The system successfully measured a signal with ten frequencies between 4.5 GHz and 5.5 GHz with a spacing of 100 MHz, achieving an accuracy of ±0.2 MHz after calibration. It also measured four frequencies separated by 1 GHz with an error of less than ±0.4 MHz. Table 2 compares the published results of various MRR, MDR, and IRR-based MFM techniques.

### 2.4. FTTM Using Sweeping Signals and Pulse Identification

**Figure 9:**
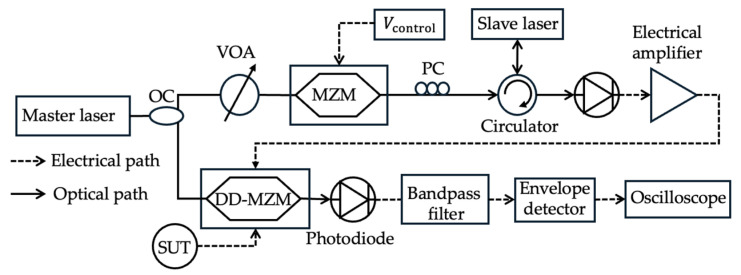
The schematic diagram of the frequency measurement system based on SBS (adapted from [69]). OC: optical coupler; PC: polarization controller; VOA: variable optical attenuator.

This subcategory includes techniques for mixing the unknown microwave signal with a broadband linear frequency-modulated (LFM) or linearly chirped signal. The frequency difference between the two signals varies linearly with time, and detecting a specific beat frequency (often selected by a narrowband filter) occurs at a time directly related to the unknown frequency. Zhou et al. [69] used a broadband triangular LFM waveform mixed with the signal under test in a dual-drive Mach-Zehnder modulator (DD-MZM) operating at the minimum transmission point (Figure 9). An oscilloscope then detects the signal comprising a pulse pair after passing through an electrical bandpass filter and envelope detector. The time delay between the pulse pair depends on the frequency of the unknown microwave signal, the bandwidth, and the properties of the LFM signal, including its central frequency, bandwidth, and temporal period. The LFM signal is achieved by modulating the optical carrier (master laser) with a triangular signal using an MZM and injecting it into a single-mode distributed-feedback semiconductor laser (slave laser). The output of the slave laser is then detected with a photodetector and fed to the DD-MZM. A measurement range of 1–39 GHz, a resolution of 20 MHz, and a measurement accuracy of ±50 MHz are achieved. Shi et al. [70] also demonstrated a 6 GHz bandwidth and a measurement resolution of 40 MHz, which was further developed to a 7 GHz bandwidth and a measurement resolution of 37.6 MHz [71]. The same group improved the technique by incorporating deep neural network (DNN)-assisted frequency estimation and demonstrated a signal measurement from 43 to 52 GHz with an average error of about 3.2 MHz [72]. Improving the technique further, Shi et al. [73] achieved a 28 GHz frequency measurement range with an average error of 9.31 MHz. Wang et al. [74] used optical sideband sweeping and intermediate frequency envelope monitoring and demonstrated a 16 to 26 GHz frequency measurement range with an average error of 7.53 MHz, with estimation errors within 11.92 MHz. The technique could distinguish two unknown signals with at least a 40 MHz frequency interval between them.

**Figure 10:**
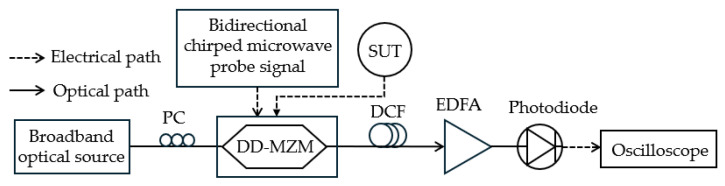
The schematic diagram of the frequency measurement system based on SBS (adapted from [75]). PC: polarization controller; DD-MZM: dual-drive Mach–Zehnder modulator; EDFA: erbium-doped fiber amplifier; DCF: dispersion compensation fiber.

Chen et al. [75] use a broadband optical signal that is intensity modulated by the unknown microwave signal and a bidirectional chirped microwave signal (Figure 10). The signal is then passed through a dispersion-compensating fiber (DCF), the dispersion of which works as a narrowband photonic filter. This filter equivalently selects specific frequency components based on the time delay induced by dispersion, converting the frequency information into the time domain. For a single unknown frequency, a pair of pulses is generated within one period of the probe signal. The unknown frequency is determined by calculating the time interval between these generated pulses. The bidirectional chirped nature of the probe signal establishes a mapping between the unknown signal’s frequency and the time difference between the pulses. A cross-correlation-based scheme is employed to overcome noise interference caused by the broadband optical source and erbium-doped fiber amplifier (EDFA). A reference signal with a single period is constructed based on the bandwidth of the photonic filter. The cross-correlation function between the measured electrical and reference signals is then calculated. This process accurately extracts the pulse positions and effectively suppresses noise interference, improving the accuracy of the time interval measurement. The system identifies single and multiple microwave frequencies within the 2 to 14 GHz range with errors less than ±3 MHz. Frequencies with a separation of 60 MHz showed blurred resolution, while a 70 MHz separation allowed for distinguishable pulses after cross-correlation.

**Figure 11:**
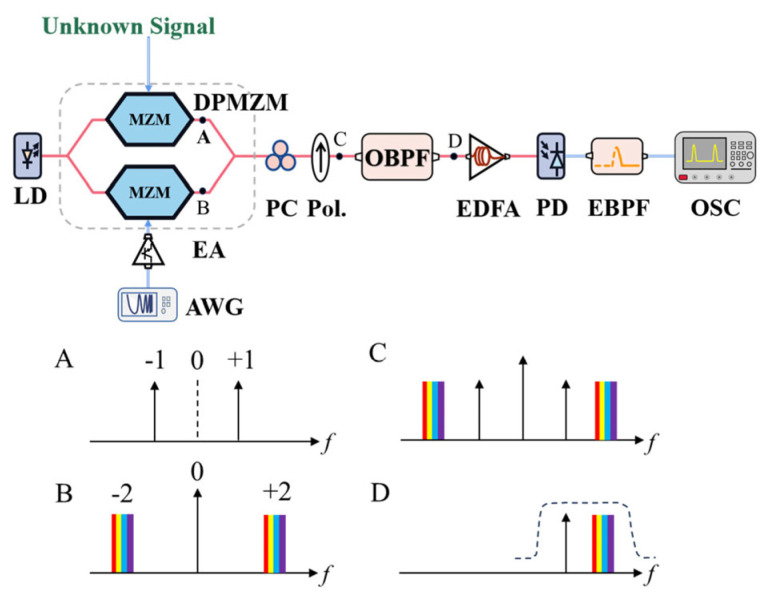
The schematic diagram of the frequency measurement system for single-tone and multi-time frequency-to-time mapping [76]. LD: laser diode; AWG: arbitrary waveform generator; EA, electrical amplifier; PC: polarization controller; Pol.: polarizer; OBPF: optical bandpass filter; EDFA: erbium-doped Fiber amplifier; PD: photodetector; EBPF: electrical bandpass filter; OSC: oscilloscope. (A–D) The spectrograms of the corresponding positions in the schematic configuration.

Xie et al. [76] utilized a DP-MZM operating in CS-SSB mode to perform single-tone and multi-time frequency-to-time mapping (Figure 11). The DP-MZM was modulated with the unknown microwave signal (*f*_i_) and a bidirectional linearly chirped microwave (LCM) signal (*f*_s_). The optical bandpass filter was set to only select the upper first-order sideband of the unknown signal and the upper second-order sideband of the bidirectional LCM signal, doubling the effective bandwidth of the LCM signal and allowing for measurement over a broader range. Mixing of these two sidebands generates a beat frequency of |2*f*_s_ − *f*_i_|, which is then detected by a photodetector. An electrical bandpass filter also only selects the beat frequency signal. By identifying the temporal position of the beating pulse, the frequency of the unknown signal (*fi*) can be accurately determined using the characteristics of the bidirectional LCM signal and the electrical bandpass filter. This technique enables an unambiguous frequency measurement without the need for a reference signal. The IFM for single-tone frequency signals achieved a measurement range of 6–16 GHz and 26–36 GHz with a measurement error of ±1 MHz. Multi-tone signal detection was also demonstrated with 50 MHz frequency resolution.

### 2.5. FTTM Combined with FTPM

**Figure 12:**
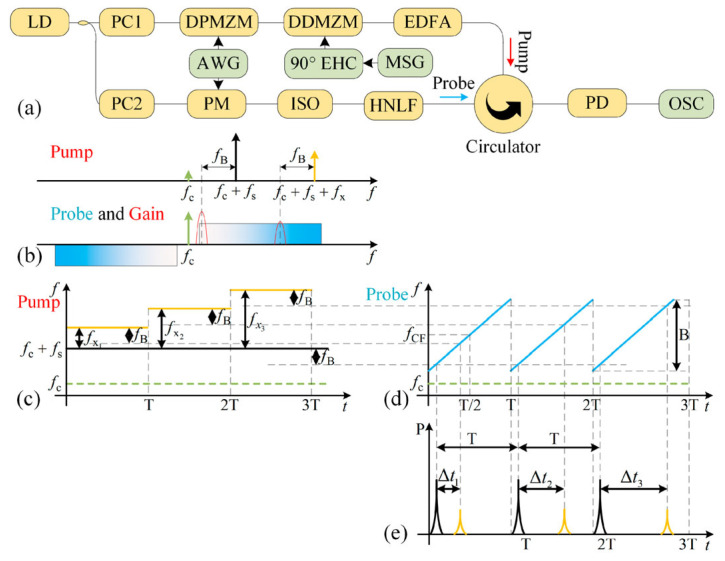
Details about the proposed scheme. (**a**) The diagram of the proposed MFM system. (**b**) The optical spectrum of the pump and probe light when the SUT is a single-tone signal. The pump wave (**c**), probe wave (**d**), and PD output (**e**) when the SUT is a step-swept signal [77].

**Figure 13:**
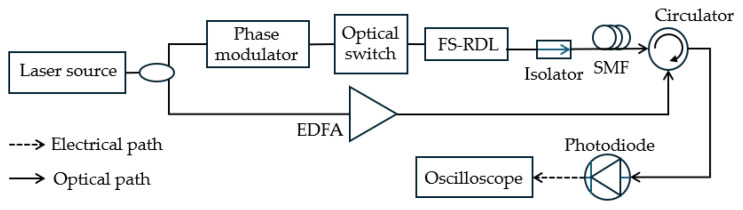
Microwave photonic signal processor based on SBS and FS-RDL [80].

While using FTPM does not allow for multi-tone MFM, combining it with FTTM can improve the measurement range and resolution. Yang et al. [77] have developed a two-step system by combining FTTM and FTPM to enable simultaneous coarse and fine measurements. As shown in Figure 12, a laser diode output is divided into two branches. The upper branch generates a pump wave through CS-SSB modulation with a chirp signal, followed by an SSB modulation with an unknown microwave frequency signal. The pump wave has a frequency-shifted carrier that works as the reference. In the lower branch, the laser is phase-modulated with a chirp signal to generate the probe wave. Both pump and probe waves propagate in an HNLF in opposite directions. The pump generates an SBS gain as it travels through the HNLF, and the probe wave becomes amplified when its frequency aligns with the Brillouin gain spectrum and is detected by a low-speed photodetector. The coarse measurement is achieved by calculating the time difference between the signal and reference pulses using the chirp rate of the sweeping signal. The fine measurement happens when a series of single-tone frequencies (probe signals) near the estimated frequency are generated based on the coarse measurement result and modulated as sidebands on the optical carrier. The gain of these probe signals depends on their position within the Brillouin gain spectrum that is created by the sidebands corresponding to the signal under test. This results in different power levels from which the precise frequency of the signal under test can be determined by analyzing the power levels of these probe signals and utilizing the narrow bandwidth and Lorentz shape of the Brillouin gain spectrum. A measurement bandwidth of 1–18 GHz with an error of less than 10 MHz and a resolution of 40 MHz is achieved. Using only phase modulation and the SBS of an SMF to perform FTTM, Nguyen et al. [78] demonstrated a multi-frequency measurement capability over a frequency range of 0.1–20 GHz, with a measurement resolution of 250 MHz. The measurement bandwidth can be extended to 90 GHz by increasing the achievable number of circulations in the frequency-shifting recirculating delay line (FS-RDL) loop, as shown in Figure 13. Huang et al. [79] simulated a 16.384 GHz measurement bandwidth and an accuracy better than 100 MHz using a fast-course measurement, and an accuracy of 20 MHz with a slow, fine measurement with a sweep rate of 0.2 GHz/µs, utilizing a two-step measurement system.

For single-stage measurements, Minasian et al. [80] further improved the technique using SBS and an FS-RDL to establish an all-optical, bias-voltage-free, optical SSB modulator (Figure 13). Controlled pulses are injected into an optical switch, which time-gates the optical signal into the FS-RDL loop. The output of the FS-RDL is then routed through an SBS-based optical filter implemented in an SMF. The time gap between the carrier pulse and the output pulses from the upper sideband is utilized to calculate the frequency of the microwave signal. Li et al. [49] also achieved a 16 GHz bandwidth with a 60 MHz resolution by jointly using three optical frequency combs, channelization, and SBS-based FTTM to extract the time-frequency information of the signal under test at different frequency intervals.

### 2.6. FTSM Using a Nonuniform Optical Frequency Comb

**Figure 14:**
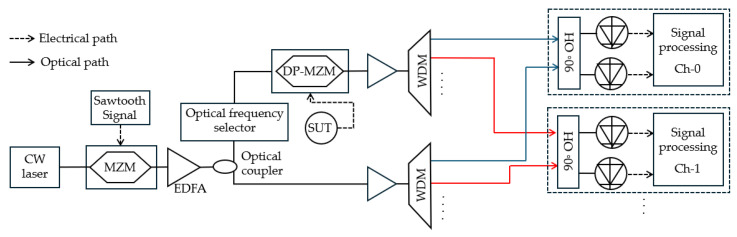
The schematic diagram of the frequency measurement system based on SBS (adapted from [35]). WDM: wavelength division multiplexing; OH: optical hybrid.

Ji et al. [35] demonstrated a broadband reconfigurable instantaneous multifrequency MFM system using a nonuniform OFC and channelization (Figure 14). A CW laser is intensity-modulated by a sawtooth signal to achieve CS-SSB modulation, generating a nonuniform OFC. The comb signal is split between two paths, one of which goes through an optical frequency selector and a DP-MZM and is modulated by the SUT. The other one passes through a wavelength demultiplexer after being amplified by an EDFA. The intensity-modulated signal with the unknown microwave signal is also channelized using a wavelength demultiplexer. The outputs of the two demultiplexers are then merged using a 90° optical hybrid coupler and detected using a pair of balanced photodetectors (BPDs). The unknown microwave signal frequency is determined by detecting the channel position in which the signal falls and the frequency that is detected within that channel. Simulation experiments show a measurement range of 0.01–70 GHz with 35 OFC lines, with an error within ±3.24 MHz. By increasing the OFC lines to 51, the measurement range is increased to 102 GHz without any changes in the error.

**Figure 15:**
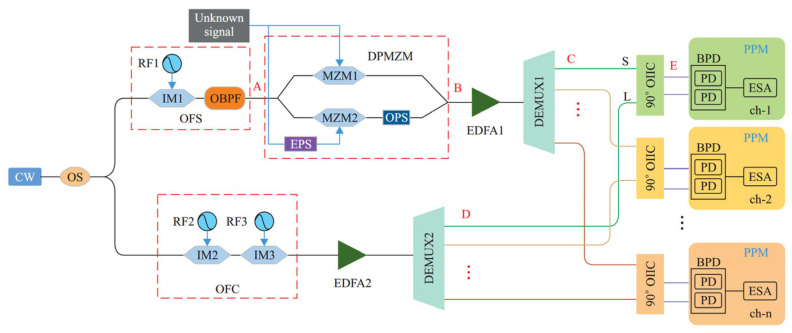
The schematic diagram based on optical frequency combs using channelization [37]. OS: optical splitter; OBPF: optical bandpass filter; IM: intensity modulators; EDFA: erbium-doped fiber amplifier; OHC: optical hybrid couplers; PPM: post-processing modules; BPD: balanced photodetector; EPS: electrical phase shifter; ESA: electrical spectrum analyzer.

Zhang et al. [37] designed a 100 GHz system to combine an optical frequency shifter with an OFC and used channelization for IFM (Figure 15). In the lower branch of the system, the original optical carrier is modulated by two intensity modulators driven by RF signals (*f*_2_ and *f*_3_) to generate a 25-line OFC with a comb spacing of *f*_3_. The optical frequency shifter (OFS) comprises an intensity modulator and an optical bandpass filter, resulting in a shift in the optical carrier frequency (*f*_c_) of a CW laser by a radio frequency signal (*f*_s_). This shifted carrier (*f*_c_ − *f*_s_) is then used as the input to the subsequent stage. The shifted carrier is then modulated by the unknown microwave frequency (*f*_xn_) in a DP-MZM operating in the carrier-suppressed upper sideband (CS-USB) modulation, resulting in *f*_c_ − *f*_s_ + *f*_xn_. The frequency of the first comb line of the OFC is designed to be the same as the shifted optical carrier frequency output from the OFS (*f*_0_ = *f*_c_ − *f*_s_). The microwave frequency-modulated signal from the upper arm and the 25-line OFC are both channelized through two separate demultiplexers, and the output of the two demultiplexes is combined with 90-degree optical hybrid couplers and detected with BPDs. With a channel bandwidth of 2 GHz, the system achieved a frequency measurement range of 0.01–50 GHz with a frequency measurement error of less than ±5 MHz. With a channel bandwidth of 4 GHz, the system’s measurement range was extended to 0.01–100 GHz, with a frequency measurement error of 5–14.6 MHz. This demonstrates the reconfigurability of the system and addresses the frequency ambiguity and mirror image blurring. Table 3 compares the published results of various FTSM-based MFM techniques.

### 2.7. FTSM Combined with FTTM

**Figure 16:**
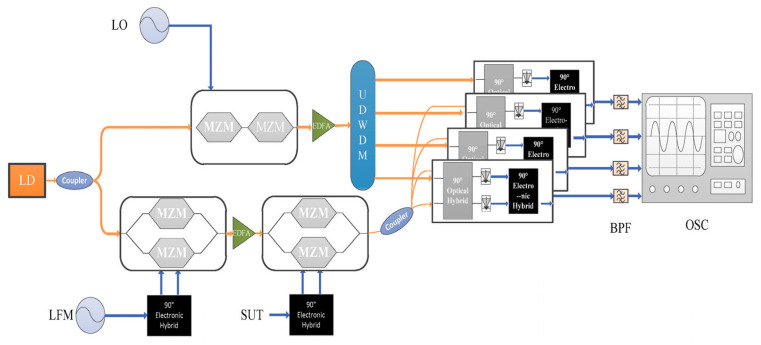
Schematic diagram of the FTTM technique combined with channelization [36]. LD: laser diode; LFM: linear frequency modulation; UDWDM: ultra-dense wavelength division multiplexer; BPF: bandpass filter; OSC: oscilloscope.

Another hybrid technique is possible by combining FTSM and FTTM. Li et al. [36] proposed and demonstrated an FTTM technique combined with channelization to expand the frequency measurement range (Figure 16). A CW laser output was split into two branches, where the upper branch goes through an OFC generator using a local oscillator and cascaded MZMs to generate a comb signal at *f*_c_, *f*_c_ + *f*_LO_, *f*_c_ + 2*f*_LO_, and *f*_c_ + 3*f*_LO_ frequencies, where *f_c_* is the laser frequency and *f_LO_* is the frequency of the local oscillator. The signals are separated using an ultra-dense wavelength multiplexer. The lower branch goes through two cascaded DP-MZMs. The first DP-MZM is modulated with two LFM signals with a π/2 phase difference biased for CS-SSB modulation, and the second DP-MZM is similarly modulated with the SUT. The modulated signal is then divided into four branches, each of which is combined with one of the upper branch signals through a photonic image rejection mixer, which down-converts the SUT in each channel followed by a 1-GHz bandpass filter generating a pulse whose time delay is related to the unknown microwave frequency. The experiment achieved a frequency measurement range of 1 GHz to 39 GHz with a frequency measurement error kept within ±20 MHz and a resolution of 10 MHz for a two-tone signal with high linearity (R^2^ = 0.99999) over the measurement range.

**Figure 17:**
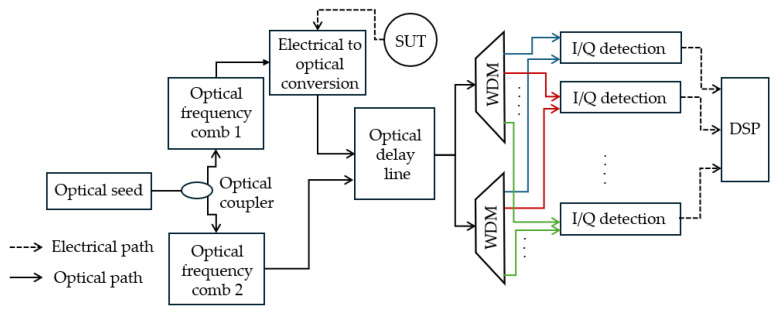
Schematic diagram of the IFM technique based on WDM and TDM (Adapted from [90]).

Li et al. [90] utilized a unique combination of wavelength division multiplexing (WDM) and time division multiplexing (TDM) (Figure 17). The technique uses dual coherent OFCs with slight detuning in comb spacing and a gated recirculating optical frequency shifter (GR-OFS) with an optical delay line that enables fast measurements of wideband microwave signals with low-bandwidth detection units. The unknown frequency signal is translated to the optical domain using an electrical-to-optical converter as sidebands of the comb lines in the upper path. After the optical delay line, the signals are fed into wavelength de-multiplexers to separate different WDM channels that are detected with I/Q coherent receivers. The mismatch in the comb spacing allows for sequential interrogation of the RF signal segments. The experimental results demonstrate up to 8 GHz instantaneous bandwidth at 1 MHz resolution with an 80 MHz RF detection unit for interrogating fast-varying and continuous RF signals with ~1 µs temporal duration.

### 2.8. FTSM Combined with FTPM

**Figure 18:**
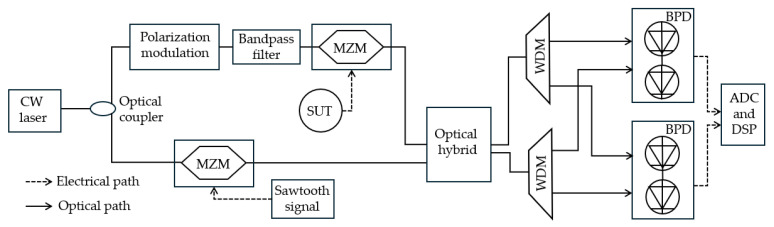
Schematic diagram of the IFM technique based on a combination of FTSM with FTPM (adapted from [83]). WDM: wavelength division multiplexer; ADC: analog-to-digital converter.

Channelization can also be combined with FTPM to enable multi-tone MFM with improved performance. Zhu et al. [83] demonstrated a multi-tone MFM system combining FTSM and FTPM (Figure 18). The FTSM happens through channelization using a non-flat OFC with adjustable comb spacing generated by sawtooth wave modulation. The FTPM is enabled by frequency identification based on the power ratios of beat frequencies within subchannels. The technique uses a 90-degree optical hybrid and BPDs for intermodulation interference cancelation. Simulation results show a frequency measurement range of 0–32 GHz using two channels. The resolution is determined by the electrical spectrum analyzer sample rate. The system noise impacts subchannel identification, possibly limiting the detection accuracy of closely spaced power ratios. The system addresses blind areas by adjusting the subchannel intervals and utilizing low-speed post-processing devices, which reduce the cost.

## 3. Chip-Based Integrated Techniques

Chen et al. [64] utilized an on-chip two-step MFM method by using an array of silicon-photonic-integrated MDRs composed of a set of MDRs with radius *r*_1_ and one MDR with radius *r*_2_ (*r*_2_ > *r*_1_). An OFC source as the optical carrier is CS-SSB-modulated by the unknown microwave signal. The system is channelized using an arrayed waveguide grating, the outputs of which are connected to an MDR notch filter with a specific resonance. The optical carriers of the OFC are designed to have a fixed wavelength relationship with these notches. Comparing the optical powers from the through and drop ports of the different MDRs in the array results in a coarse estimation of the microwave signal’s frequency. The second step utilizes a single add-drop MDR with a radius of *r*_2_, with a much narrower transmission notch compared to the smaller MDRs used for the coarse measurement. A tunable laser source (TLS) is CS-SSB modulated by the same unknown microwave signal and is rapidly tuned to position the optical sideband of the microwave signal onto the steep right side of the resonance notch of the larger MDR. The microwave signal frequency is then finely measured by monitoring the ACF resulting from the optical power ratio between the through and drop ports of the larger MDR. A proof-of-concept experiment using a 6-μm radius MDR with a 3-dB bandwidth of 21.17 GHz for coarse measurement and a 10-μm radius MDR with a 3-dB bandwidth of 4.50 GHz for fine measurement demonstrated a frequency measurement range from 1.6 to 40 GHz. Coarse measurements showed measurement errors around ±200 MHz in the frequency range of 6 to 16.2 GHz. Fine measurements achieved errors less than ±50 MHz in the frequency ranges of 4 to 8.8 GHz and 13 to 19 GHz. In the frequency ranges of 23 to 29 GHz and 32 to 38.6 GHz, measurement accuracies of ±60 MHz and ±45 MHz, respectively, were achieved.

Yao et al. [91] demonstrated a dual-modality technique combining FTTM and FTPM on a highly integrated single silicon-on-insulator (SOI) chip with a size of 3.8 mm × 2.2 mm and a weight of 6 g, with an estimated total power consumption of 3.52 W. A CW laser is intensity-modulated with a DP-MZM to generate a CS-SSB signal and split it into two paths, each of which supports one of the modalities for frequency measurement. One path utilizes a thermally tunable MRR running with a sawtooth voltage, creating a scanning filter employing FTTM. Output temporal pulses whose time corresponds to the specific frequencies present in the input signal can be observed when the MRR resonance aligns with the sidebands of the input microwave frequencies. The second path implements FTPM using three cascaded MRRs functioning as a reconfigurable MPF for selecting the frequency band of interest. The MRRs are followed by an asymmetric Mach-Zehnder interferometer and a 1 × 2 multimode interferometer enabling an ACF using two on-chip photodetectors. A pre-calibrated ACF curve is then used to identify the unknown frequency. This technique can identify single-frequency, multiple-frequency, chirped, and frequency-hopping signals. It also can discriminate instantaneous frequency variations within frequency-modulated signals. The system achieved a frequency measurement range of 10 to 20 GHz and a measurement error for single-frequency signals of approximately 409.4 MHz using FTTM and 483.8 MHz using FTPM. For multi-tone signal measurement, a frequency resolution of about 1 GHz with an error of less than 510 MHz for each tone was achieved. Chirped microwave signals centered at 15 GHz with bandwidths of 4 and 6 GHz and frequency-hopping signals with different frequency steps with a measurement error of around 166.9 MHz were identified. Table 4 compares the published results of various FTTM-based frequency measurement techniques.

## 4. Time-Stretch IFM

**Figure 19:**
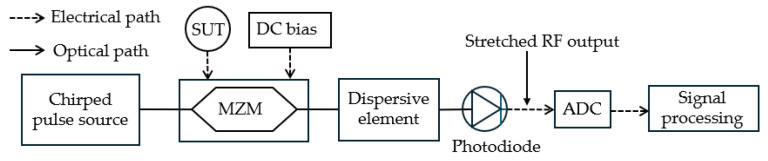
Schematic diagram of the IFM technique based on time stretch through dispersive fiber (adapted from [33]). ADC: analog-to-digital converter.

Many of the traditional techniques use CW lasers for microwave photonics and IFM. While CW laser diodes are compact and cost-efficient, using mode-locked lasers with ultrashort pulses at high repetition rates to provide a broadband pulse spectrum has its own advantages that are not attainable with CW lasers. In combination with a dispersive element, mode-locked lasers enable the simultaneous measurement of multiple frequencies and amplitudes with high accuracy over an ultrawide frequency bandwidth using lower-bandwidth electronic devices. Lam et al. [33] demonstrated the technique using a broadband optical pulse that is chirped by propagating through a dispersive fiber which maps frequency to time (Figure 19). The chirped pulses are intensity modulated by the unknown microwave signal, after which they pass through a second dispersive fiber that linearly stretches the pulses in time. This operation effectively compresses the analog bandwidth by a stretch factor that depends on the dispersion parameters of the dispersive fibers. An analog-to-digital (ADC) converter digitizes the time-stretched microwave signal with a highly effective sampling throughput, after which DSP and fast Fourier transform are applied to spectrally analyze short real-time segments of the signal. This enables simultaneous measurements of multiple frequencies and amplitudes. An ultra-wide instantaneous bandwidth is achieved from 5 to 45 GHz with a frequency estimation error of ±125 MHz and an RMS error of 97 MHz across the entire 40 GHz range, measuring multiple frequencies at the same time without the need for additional hardware or cascading filters. The frequency resolution can be tuned by adjusting the first dispersive fiber length. Compared to traditional IFM techniques, time-stretch reduces hardware complexity, and back-end DSP can be implemented in real-time, achieving ultra-wideband measurements with high accuracy for the simultaneous measurement of multiple signals.

**Figure 20:**
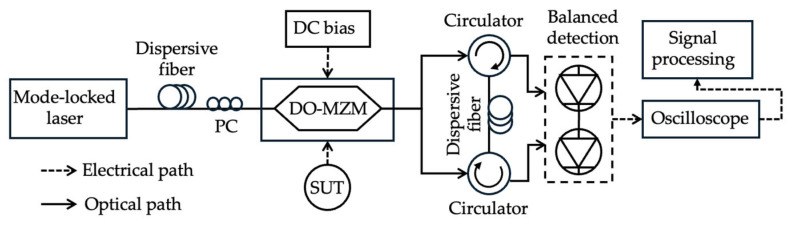
Schematic diagram of the ultra-wideband IFM technique. PC: polarization controller; DO-MZM: dual output MZM (adapted from [34]).

Bai et al. [34] also demonstrated an ultra-wideband IFM using a dual-output MZM with two complementary optical outputs with a 180-degree phase shift (Figure 20). Both outputs travel through the same dispersive fiber in opposite directions and are eventually detected with a BPD. The BPD provides differential detection of the two time-stretched (chirped) signals, suppressing pulse-to-pulse fluctuations in the laser and eliminating the distortion of the measured SUT, which is typically caused by the non-uniform spectrum of the pulsed laser source. A 3–20 GHz frequency range was achieved with a frequency resolution of 660 MHz and a measurement error of ±380 MHz.

**Figure 21:**
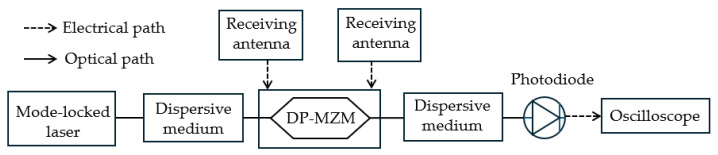
The schematic diagram of the simultaneous AOA and frequency measurement system based on time-stretch (adapted from [92]).

Ding et al. [92] demonstrated a simultaneous angle-of-arrival (AOA) and frequency measurement system. Pulses of a mode-locked laser are stretched in time by passing through a linearly chirped fiber Bragg grating (LC-FBG), creating chirped optical pulses (Figure 21). The RF signals are received by two antennas with distance *d*, resulting in a time delay and a phase difference that depends on the AOA of the microwave signal. The two received RF signals are fed into the two sub-MZMs of the DP-MZM. The interference signal of the DP-MZM maps the AOA-dependent phase difference to the power of the interfered optical sidebands. A second LC-FBG is used to compress the optical pulses in the time domain. This process maps different frequency components of the unknown RF or microwave signal to different time positions of the output electrical pulses, hence realizing FTTM. For each frequency component, the output electrical signal consists of two pulses with a time interval proportional to the unknown frequency and amplitudes related to the AOA-dependent phase difference. The system has the capability to measure muti-tone signals because different frequencies are mapped to different time intervals, making the output pulses independent in the time domain. An AOA measurement from −70° to 70° was achieved with a measurement error within ±2.5°, and a frequency measurement from 5 to 15 GHz was achieved with a measurement error within ±12 MHz.

## 5. Application of Novel Fibers in Microwave Photonics

**Figure 22:**
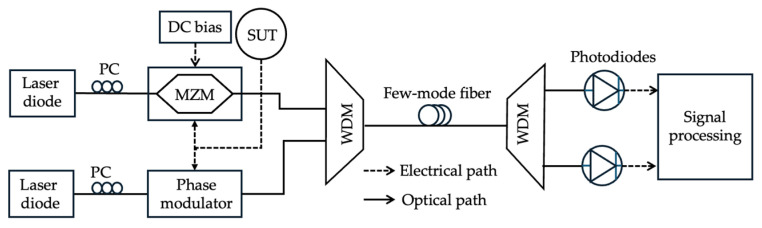
The schematic diagram of a frequency measurement system based on FMF (adapted from [87]). PC: polarization controller.

Recently, FMFs have been utilized for microwave photonic systems, replacing SMFs due to their ability for spatial division multiplexing. Wen et al. [98] first used an FMF to enhance the performance of fiber-based microwave photonic links (Figure 22). Using the FMF instead of the SMF addressed the power-handling limitations due to the FMF’s higher nonlinear threshold resulting from a larger mode field area. Additionally, the different modes of the FMFs enable spatial division multiplexing due to the walk-off between different modes or mode groups. Transmitting light through different spatial modes of the FMFs enables greater control over nonlinearities and modal delays. Nickel et al. [99] demonstrated the utilization of an FMF as a microwave photonic impulse response filter. The differential mode group delay (DMGD) between different fiber modes is used as distinct optical delay lines. This eliminates the need to use multiple discrete SMF delay lines. When a CW single-wavelength laser modulated by the microwave signal is coupled into different modes of the FMF using mode-selective multiplexers, the DMGD causes different modes to propagate at different speeds. This phenomenon results in differential time delays necessary for the filter. The different modes of the FMF can be excited either through offset splicing with an SMF or through individual ports of a mode-selective multiplexer known as photonic lanterns. While using FMFs in microwave photonic links seems to simplify the system architecture, the mode coupling and modal crosstalk might limit its application [100]. Nickel et al. [100] demonstrated that in a balanced microwave photonic link using 100 m-long FMFs with multiple linearly polarized (LP) modes, the LP_02_ + LP_01_ mode pair exhibited the best overall performance and stability, while the LP_11a_ + LP_01_ mode pair exhibited the least stable pair of modes to use.

Zhao et al. [87] used an FMF-based MPF for IFM in a two-tone multiplexed system leveraging FMF’s DMGD. A hybrid system of intensity and phase modulation was used for this study, where one CW laser was intensity-modulated and one was phase-modulated by the same unknown microwave frequency signal. The signals were combined and sent into an FMF that supports two spatial LP modes, LP_01_ and LP_11_. Offset splicing excites both modes, which are then delayed due to the inherent fiber DMGD. The differential time delay between the two modes creates an optical photonic filter for each wavelength. The filters have complementary frequency responses since one wavelength is intensity-modulated while the other is phase-modulated. One of the filters acts as a lowpass filter, while the other one has bandpass characteristics. A wavelength division multiplexer separates the two wavelength channels, and an ACF is calculated based on the power ratio of the two wavelength channels. The ACF is related to the ratio of the frequency response of two MPFs and is monotonically related to the unknown frequency. Using a calibration ACF curve, the instantaneous frequency of the unknown microwave signal is determined through comparison. A frequency measurement range of 0.5–17.5 GHz is achieved with an average measurement accuracy of ±0.2 GHz across the entire measurement range. The measurement range of this technique is influenced by the FSR of the two MPFs. This FSR is determined by the DMGD between the two modes of the FMF, the fiber length, and the overall delay between the two modes. Using FMF and mode division multiplexing eliminates the need for multiple discrete delay lines and simplifies the system architecture. Additionally, the complementary frequency responses of the pair of MPFs due to phase and intensity modulation result in an ACF with a large slope that enhances the IFM resolution.

While using standard commercially available FMFs makes implementing FMF-based IFM more practical, designing and fabricating new FMFs specifically for microwave photonics applications is also useful. Zhao et al. [101] introduced a novel technique using a specially designed FMF with a uniform DMGD between its propagating modes. The specially designed FMF is used as a delay line for microwave photonics finite impulse response filters. The fiber supports LP_01_, LP_11_, LP_02_, and LP_31_ modes with a uniform DMGD between adjacent modes across the entire C-band. The different modes propagating in the fiber experience a uniform time delay difference with the adjacent modes, and this difference is relatively constant across the C-band between 1530 nm and 1565 nm. This uniformity generates a multi-tap delay line that is required for implementing finite impulse response filters. This fiber has the potential to significantly simplify the system and reduce its complexity compared to traditional multi-fiber or multi-source approaches. Nazemosadat et al. designed [102] and demonstrated [103] a reconfigurable MPF based on a specially designed and fabricated double-clad step-index FMF operating as a tunable true time delay line. The FMF is designed to have evenly spaced incremental chromatic dispersion values among its five spatial modes: LP_01_, LP_11_, LP_21_, LP_31_, and LP_41_, which allow for tunable delay characteristics. The tunable operation of the FMF is achieved by sweeping the optical wavelength of the laser. The differential chromatic dispersion and group delay between adjacent modes must be constant to achieve continuous tunability.

**Figure 23:**
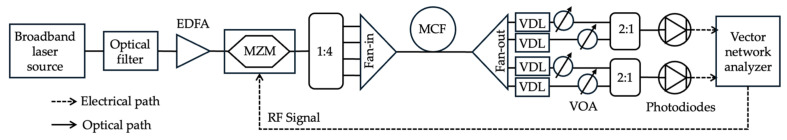
The schematic diagram of a frequency measurement system based on heterogeneous MCF (adapted from [39]). VDL: variable delay line; VNA: vector network analyzer: VOA: variable optical attenuator.

In addition to FMFs, multicore fibers (MCFs) can also be utilized for microwave photonics applications. Nickel et al. [104] utilized a four-core MCF to construct a balanced analog photonic link. Although insertion losses at the spatial multiplexers reduce the optical power, requiring higher input power compared to their SMF counterparts, MCFs could be a viable single-fiber alternative to SMF-balanced links. Nazemosadat et al. [39] presented an MFM scheme based on a heterogeneous MCF with seven cores, four of which were utilized for the measurement (Figure 23). Each core of the MCF had a different differential group delay (DGD), and two distinct two-tap MPFs were constructed by combining and detecting the outputs of two different pairs of these four cores. The FSR of the filters is determined by the DGD between the two cores in each pair. The ratio between the microwave power detected at the output of these two filters determined the ACF, which is a function of the unknown microwave frequency and the DGDs of the two filter pairs. A measurement range of 0.5–40 GHz, resolution of ±71 MHz, and RMS error value of 44 MHz were achieved.

## 6. Photonic Compressive Sensing of Microwave Signals

**Figure 24:**
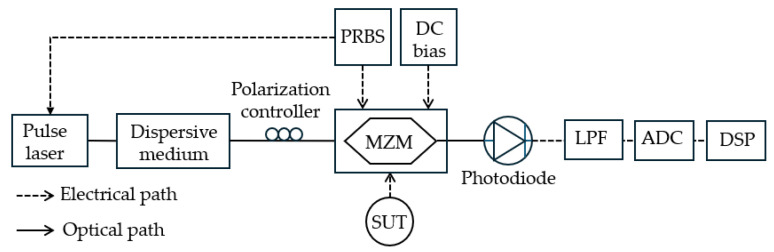
The schematic diagram of a frequency measurement system based on compressive sensing (adapted from [42]). LPF: longpass filter; PRBS: pseudo-random binary sequence.

Photonic compressive sensing (PCS) has emerged as a powerful technique for real-time, wideband, and high-resolution for the MFM. Dai et al. [42] presented a novel PCS scheme for microwave signals utilizing optical pulse random mixing, significantly enhancing both the compression ratio and frequency range compared to traditional CW systems. By addressing the non-ideal characteristics associated with pseudo-random binary sequences (PRBS)—such as sloped edges and amplitude jitter, the proposed method achieves more ideal compression, resulting in improved accuracy in signal recovery. In their proof-of-concept experiments, the authors demonstrated the successful recovery of dual-tone microwave signals within the 4 GHz range at a compression ratio of 20. Furthermore, single-tone signals beyond the first Nyquist zone—at frequencies of 7.17 GHz, 11.26 GHz, and 14.6 GHz—were accurately reconstructed with a remarkable compression ratio of 40. The innovative use of narrow-width optical pulses with high harmonic components allows for effective wideband down conversion, thereby extending the system’s operating frequency range. The experimental configuration, shown in Figure 24, mixes microwave signals with optical pulses (1550 nm wavelength, 25 ps pulse width, and 8 GHz repetition rate) for effective compression and recovery. The optical pulses are injected into the DD-MZM after passing through a polarization controller and an optical time delay line to align with the PRBS signal. The peak-to-peak voltage of the PRBS signal was amplified to match the DD-MZM’s half-wave voltage to achieve bipolar random mixing. The photodetector output was filtered through a longpass filter, and digitized by the ADC converter (a 50 GHz sampling oscilloscope). After digitization, the recovered signal is processed using DSP algorithms to reconstruct the original microwave signal. The performance is assessed through experiments with various signals, including dual-tone signals with components at 500 MHz and 3.65 GHz.

## 7. Recent Advances in AI

The integration of ML techniques into IFM systems has emerged as a significant advancement. Traditional methods of IFM often face limitations such as latency, sensitivity to electromagnetic interference, and restricted measurement ranges. However, recent developments indicate that ML can enhance the performance of IFM systems, making them more efficient and accurate. One of the primary advantages of employing ML is its ability to process and analyze large datasets rapidly, which is crucial for identifying dynamic microwave signals with time-varying frequencies. For instance, Liu et al. [105] leverage ML techniques in IFM to improve measurement accuracy and response time and demonstrate the potential for real-time applications in environments where fast frequency identification is essential. Shi et al. [38] implemented a stacking regression method that combines outputs from multiple ML models, including support vector regressor (SVR), K-nearest neighbors (KNN) regressor, polynomial regressor (PR), and random forest regressor (RFR), to optimize the ACF derived from FTPM. The system employs a polarization division multiplexed emulator to adjust the dispersion group delay of signals, enabling flexible measurement bandwidths. Experimental results revealed significant improvements in measurement accuracy, with reductions in maximum and average measurement errors for bandwidths of 2 GHz and 4 GHz. The system is robust against differential mode noise, particularly polarization fluctuations, making it suitable for high-demand applications in modern radar and electronic warfare systems. The findings suggest that integrating ML with photonics could offer effective solutions for the challenges faced in high-frequency microwave measurements.

**Figure 25:**
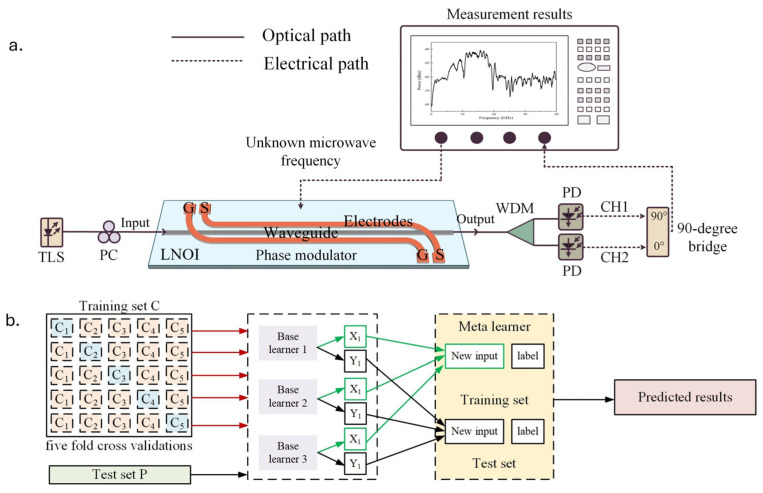
(**a**) Schematic of the IFM system consisting of lithium niobate on an insulator (LNOI) phase modulator, tunable laser source (TLS), polarization controller (PC), wavelength division multiplexer (WDM), photodiode (PD), (**b**) stacking ensemble machine learning model architecture [107].

Jia et al. [106,107] and Chen et al. [108] presented an IFM technique based on a stacked integrated ML model to enhance performance that consists of multiple base learners, which are various ML algorithms that contribute to the prediction process (Figure 25). These algorithms work together to handle different aspects of the data and improve overall performance. A higher-level classifier, known as the meta (Tier-2) classifier, is then used to combine the outputs from the base (Tier-1) classifiers [109]. This meta-learner strategically analyzes the predictions from the various base models to produce a more accurate measurement output that the hardware system cannot provide. The measurement system effectively spanned a wide frequency range from 0 to 40 GHz. The system maintained its functionality even with low-RF input power levels down to −30 dBm, showing its robustness under less-than-ideal conditions. The RMS error was measured at 62.06 MHz for these lower input levels, reflecting consistent performance.

Furthermore, different neural network techniques like deep neural networks (DNNs), generative adversarial networks (GANs), and convolutional neural networks (CNNs) have been implemented for MFM [72,110]. Liu et al. [9] explored the integration of DNNs into photonic systems, employing a CS-DSB modulation technique for frequency estimation. At first, the RF signal was modulated onto an optical carrier, and the optical output was processed through complementary triangular spectral responses, enabling precise optical power measurements that correlate with the RF frequency. The resulting dataset, including key parameters such as RF frequency, FSR, extinction ratio, and optical power measurements, was divided into training (90%), validation (5%), and testing (5%) subsets to ensure robust model evaluation. A DNN with three hidden layers (10–20–5 neurons) was constructed to implement a Levenberg–Marquardt optimization algorithm for model training. The DNN demonstrated exceptional performance in predicting frequencies with an RMS error of merely 1.1 MHz over a frequency sweep of 14 GHz, achieving an R^2^ value of 99.94% during validation. When tested with new input data, the prediction error remained under 50 MHz, confirming the system’s effectiveness under varying operational conditions. Jabin et al. [8] further improved this model by employing generative adversarial networks (GANs), which consisted of a generator based on a recurrent neural network with long short-term memory layers and a discriminator using CNNs. This framework significantly reduces the number of experimental datasets required from 6000 to just 75, while augmenting this limited dataset to generate 5000 synthetic datasets for training. The methodology substantially reduces the frequency measurement error from 50 MHz to 5 MHz, showcasing a ten-fold enhancement in accuracy.

## 8. Current Challenges and Future Prospects

While FTTM and FTSM techniques are pivotal in MFM systems, they exhibit notable limitations that impact their efficacy and application across various scenarios. The FTTM techniques present significant advantages in terms of resolution and measurement speed, particularly for broad bandwidths. However, their operational constraints often lead to lengthy measurement times when applied to multiple frequency signals. While FTTM can handle a range of frequencies up to 90 GHz with a resolution of 250 MHz [78], the technique is hampered by its limited measurement time window and dependence on the bandwidth of the optical components. This bottleneck restricts its utility in high-speed applications where instantaneous measurements are necessary, as extended measurement times can lead to errors in time-critical scenarios [97]. Furthermore, the resolution of FTTM is fundamentally constrained by the performance of the optical delay lines and modulators involved, which can ultimately limit the maximum frequency measurable within a given testing setup [15]. On the other hand, FTSM translates frequency data into spatial parameters, which can enhance certain aspects like sensitivity and robustness. However, FTSM approaches often require complex spatial configurations that can complicate the design and integration of measurement systems [35,36,37]. While this advanced technique enhances sensitivity, it relies on multiple filters, which can affect overall system complexity and performance under practical conditions. Moreover, although FTSM can provide certain advantages in terms of robustness against interference, it often cannot match the instantaneous measurement capabilities that FTPM systems offer, which are crucial for real-time applications. Additionally, the transition from frequency measurement in the electronic to photonics domain, while innovative, presents challenges such as decreased efficiency in uncalibrated environments and potential inaccuracies in frequency interpretation due to the need for robust calibration techniques [15].

The future prospects of photonic MFM techniques present an innovative and multi-faceted domain, especially when considering the implications of chip-scale integration, advanced signal processing techniques, and ML applications. These key aspects are critical for establishing tighter integration, enhancing performance, and broadening application scopes across various fields, including telecommunications, radar systems, and sensor technologies. Chip-scale integration plays a pivotal role in the advancement of microwave photonic systems. Recent advancements, as discussed by Liu et al. [6], emphasize the synergy of integration technologies and optimization techniques, which could lead to all-integrated, high-performance microwave photonic devices. Such integration not only reduces the physical footprint but also enhances device performance through improved power efficiency and stability. The realization of fully chip-scale microwave photonic systems, as highlighted by Tao et al. [40], provides a significant impact across a range of applications, including optical-to-electrical conversions and high-precision oscillators. The use of integrated solutions can lead to better key performance metrics such as phase noise and power efficiency, which are essential for modern high-speed applications. Moreover, signal processing in microwave photonic systems benefits significantly from integration. The development of programmable photonic integrated circuits (PICs) for signal processing has emerged as a vibrant area of research. Zhang et al. [111] showcase the potential of integrated field-programmable disks for various applications, including beamforming and dynamic filtering. Such PICs allow for the flexible and reconfigurable processing of microwave signals, enhancing versatility and functionality in addressing specific application demands. Furthermore, the ability to create reconfigurable microwave photonic processors on-chip [112] paves the way for scalable, multifunctional devices that can be adapted for various tasks while maintaining performance integrity.

Challenges remain in realizing the full potential of these advancements, particularly in mitigating noise performance while achieving high reconfigurability. The integration of diverse functionalities on a single chip also demands robust design methodologies to handle electromagnetic interference and other operational constraints inherent in high-frequency applications [5,7]. Advanced signal processing, including ML and deep learning, also enhances measurement accuracy in photonic systems. Creating robust methods against environmental disturbances involves using advanced materials and packaging for photonic components, along with real-time DSP for maintaining accuracy amid temperature fluctuations and vibrations. Balancing measurement range and resolution requires innovative approaches, such as optimizing FTTM and MPFs, enhancing optical components, and exploring new modulation and detection techniques. To enhance practical applications, frequency measurement systems must simplify and scale, reducing component complexity and improving modularity such as utilizing FMFs and MCFs. Future advancements will involve integrating photonics, signal processing, and ML to address challenges in system complexity, environmental sensitivity, and measurement limitations, leading to more precise, reliable, and scalable solutions.

## 9. Conclusions

Despite the advancements in frequency measurement technologies, several challenges remain. Environmental factors, such as temperature fluctuations and electromagnetic interference, continue to affect measurement stability and accuracy. Developing methods that are robust against these disturbances is crucial for further progress. Prospects in frequency measurement involve the continuous integration of advanced materials, improved signal processing techniques, and artificial intelligence. These advancements aim to overcome existing limitations and achieve unprecedented levels of precision and reliability. For instance, developing integrated microwave photonic systems and applying ML for real-time data analysis are promising areas for future research. In conclusion, the evolution of frequency measurement methods from traditional mechanical and electronic approaches to modern optical and microwave techniques demonstrates significant progress in this field. Each technological breakthrough builds upon the limitations of earlier methods, introducing innovative solutions that enhance precision, stability, and applicability.

## Figures and Tables

**Table 1:** Comparison of different SBS and OEO-based filtering multi-tone MFM methods.

Ref.	Technique	Range (GHz)	Resolution	Accuracy/Error
[43]	SBS-based FTPM and ACF	9–38	25 MHz	<±1 MHz
[47]	SBS-based FTTM, scanning system	0–30	40 MHz	≤10 MHz
[48]	SBS-based FTTM, scanning system	0.1–20	18 MHz	≤5 MHz
[15]	SBS-based FTTM	6–18	40 MHz	1 MHz
[49]	SBS-based channelized FTTM	0–12	60 MHz	-
[45]	FTTM, FDML OEO, scanning system	0–15	60 MHz	≤±60 MHz
[46]	FTTM, FDML OEO, scanning system	1–16	-	≤±70 MHz
[50]	ACF based on SBS notch filter	0.01	-	±250 kHz
[51]	FTTM, OEO and ring resonator	0–20	200 MHz	≤±100 MHz

**Table 2:** Comparison of different MRR, MDR, and IRR-based filtering multi-tone MFM methods.

Ref.	Technique	Range (GHz)	Resolution	Accuracy/Error
[52]	FTTM Scanning system, Si-MRR	5–30	5 GHz	≤±510 MHz
[54]	FTTM Ring resonator and heterodyne detection	10	1 MHz	±0.04 MHz
[55]	Si-MDR bandpass filter	7–25	-	-
[56]	As_2_S_3_ SBS notch filter	0–30	33–88 MHz	-
[57]	SiO_2_ microsphere notch filter	15	2.2 MHz	-
[58]	Si-MRR notch filter	6–19	-	-
[59]	aluminum nitride MRR notch filter	4–25	-	-
[60]	Si-MRR notch filter	1.0–8.3	150 MHz	-
[61]	Si-MRR bandpass filter	2.0–18.4	170 MHz	-
[62]	Si waveguide Bragg grating	0–32	-	773 MHz (rms)
[63]	Si-MRR, ACF	3–19	±0.5 GHz	500 MHz
[64]	Si-MDR, ACF	1.6–40	-	60 MHz
[65]	Si-MRR, FTTM	1–30	375 MHz	237.3 MHz (rms)
[66]	Si-MRR, ACF	14–25	-	200 MHz
[67]	Si-MRR, FTTM	2-18	20 MHz	-
[68]	Si-MDR, tunable, ACF frequency resolution dependent	10	-	100 MHz
[17]	Si-MRR, tunable, ACF frequency resolution independent	5–20	80 dB/GHz	47.2 MHz
[53]	High Q-factor Magnesium fluoride MDR	14.25–17.25	-	10 MHz

**Table 3:** Comparison of multi-tone MFM methods based on the FTSM technique.

Ref.	Technique	Range (GHz)	Resolution	Accuracy/Error
[81]	FTSM based on OFC	0.5–39.5	0.5 GHz	±500 MHz
[82]	FTSM based on OFC and photonic channelization receiver	<70	-	-
[83]	FTSM and FTPM	0–32	-	-
[84]	FTSM based on optical beating between CS-DSB signal and an OFC	2–12	-	2 MHz
[85]	FTSM with a channelizer consisting of OFC and OFS	1–72	-	<±2 MHz
[37]	FTSM with channelizer consisting of OFC and OFS	0.01–50 0.01–100	10 MHz	<±5 MHz 5–14.6 MHz
[86]	FTSM using phase deference	5–20	20 MHz	±10 MHz
[35]	FTSM with nonuniform OFC	0.01–70 0.01–102	-	<±3.24 MHz <±3.24 MHz
[87]	FTSM using an FMF	0.5–17.5	-	±200 MHz
[88]	FTSM based on photonic crystal nanocavities	9–19	-	±150 MHz
[89]	FTSM based on polarization interference	4.4–8.7	≤200 MHz	<±200 MHz

**Table 4:** Comparison of different FTTM techniques for IFM and MFM.

Ref.	Technique	Range (GHz)	Resolution	Accuracy/Error
[92]	FTTM and angle-of-arrival to power	5–15	-	<±12 MHz
[93]	FTTM based on a channelized receiver using an optical comb	0.5–11.5	-	±500 MHz
[94]	FTTM based on OFC	2.2–20	-	<2 MHz
[52]	FTTM based on silicon MRR	0–25	5 GHz	<±510 MHz
[54]	FTTM based on IRR	0–10	<1 MHz	<±0.4 MHz
[75]	FTTM and cross-correlation	2–14	-	<±3 MHz
[15]	FTTM based on SBS	6–18	18 MHz	<±1 MHz
[95]	FTTM using a dispersive medium	15–45	12.5 GHz	±1560 MHz
[78]	FTTM based on frequency shifting recirculating delay line loop	0.1–20	0.25 GHz	200 MHz
[96]	FTTM using fiber Bragg grating pair composed Fabry–Pérot (FBG-FP)	1–15	0.2 GHz	90 MHz
[79]	FTTM based on two-step MFM	0–12	700 MHz	100 MHz
[74]	FTTM using optical sideband sweeping	16–26	40 MHz	7.53 MHz
[97]	FTTM based on equivalent frequency sampling	30–33	46 MHz	<6.5 MHz
[77]	FTTM combined with FTPM	1–18	40 MHz	10 MHz

## References

[1] Ivanov A., Morozov O., Sakhabutdinov A., Kuznetsov A., Nureev I. (2022). Photonic-Assisted Receivers for Instantaneous Microwave Frequency Measurement Based on Discriminators of Resonance Type. Photonics.

[2] Zhou Y., Wang L., Liu Y., Yu Y., Zhang X. (2023). Microwave Photonic Filters and Applications. Photonics.

[3] Zou X., Lu B., Pan W., Yan L., Stöhr A., Yao J. (2016). Photonics for microwave measurements. Laser Photonics Rev..

[4] Bui L.A. (2020). Recent advances in microwave photonics instantaneous frequency measurements. Prog. Quantum Electron..

[5] Daulay O., Botter R., Marpaung D. (2020). On-chip programmable microwave photonic filter with an integrated optical carrier processor. OSA Contin..

[6] Liu Y., Marpaung D., Eggleton B.J., Choudhary A. (2018). High-performance chip-assisted microwave photonic functionalities. IEEE Photonics Technol. Lett..

[7] Romero Cortés L., Onori D., Guillet de Chatellus H., Burla M., Azaña J. (2020). Towards on-chip photonic-assisted radio-frequency spectral measurement and monitoring. Optica.

[8] Jabin M.A., Fok M.P. (2022). Data augmentation using a generative adversarial network for a high-precision instantaneous microwave frequency measurement system. Opt. Lett..

[9] Liu Q., Gily B., Fok M.P. (2021). Adaptive Photonic Microwave Instantaneous Frequency Estimation Using Machine Learning. IEEE Photonics Technol. Lett..

[10] Wang C., Yao J. (2013). Ultrahigh-resolution photonic-assisted microwave frequency identification based on temporal channelization. IEEE Trans. Microw. Theory Tech..

[11] Huang C., Chan E.H.W., Hao P. Frequency-To-Space Mapping based Instantaneous Frequency Measurement System with Improved Accuracy and Resolution. Proceedings of the 2023 International Topical Meeting on Microwave Photonics (MWP).

[12] Zhang J., Ma D., Li P. (2025). Multifrequency measurement system with no frequency ambiguity based on two frequency-space mappings. Opt. Eng..

[13] Xie X., Dai Y., Xu K., Niu J., Wang R., Yan L., Lin J. (2012). Broadband photonic RF channelization based on coherent optical frequency combs and I/Q demodulators. IEEE Photonics J..

[14] Zou X., Li W., Pan W., Yan L., Yao J. (2013). Photonic-assisted microwave channelizer with improved channel characteristics based on spectrum-controlled stimulated Brillouin scattering. IEEE Trans. Microw. Theory Tech..

[15] Liu J., Shi T., Chen Y. (2021). High-Accuracy Multiple Microwave Frequency Measurement with Two-Step Accuracy Improvement Based on Stimulated Brillouin Scattering and Frequency-to-Time Mapping. J. Light. Technol..

[16] Zhang B., Zhu D., Chen H., Zhou Y., Pan S. (2020). Microwave frequency measurement based on an optically injected semiconductor laser. IEEE Photonics Technol. Lett..

[17] Song S., Chew S.X., Nguyen L., Yi X. (2021). High-resolution microwave frequency measurement based on dynamic frequency-to-power mapping. Opt. Express.

[18] Zhi K., Huang C., Chan E.H.W., Hao P., Wang X. (2024). All-optical instantaneous RF signal frequency measurement system based on a linear ACF with a steep slope. Appl. Opt..

[19] Shi J., Zhang F., Ben D., Pan S. (2019). Photonics-Based Broadband Microwave Instantaneous Frequency Measurement by Frequency-to-Phase-Slope Mapping. IEEE Trans. Microw. Theory Tech..

[20] Huang C., Chan E.H.W. (2022). Microwave frequency measurement based on a frequency-to-phase mapping technique. Opt. Lett..

[21] Wang T., Yang Y., Xie S., Luan S., Li Y. (2024). Frequency-Phase-Mapping Method in Microwave Frequency Measurement With Single Optical Frequency Comb. IEEE Sens. J..

[22] Gao J., Ma J. (2023). Photonic instantaneous microwave frequency measurement based on frequency-phase mapping with high precision. Appl. Opt..

[23] Wang D., Du C., Yang Y., Zhou W., Meng T., Dong W., Zhang X. (2020). Wide-range, high-accuracy multiple microwave frequency measurement by frequency-to-phase-slope mapping. Opt Laser Technol..

[24] Kumar C., Nadeem M.D., Raghuwanshi S.K., Kumar S. (2024). Recent Advancement in Microwave Photonics Sensing Technologies: A Review. IEEE Sens. J..

[25] Zhang Y., Pan S. (2018). Broadband Microwave Signal Processing Enabled by Polarization-Based Photonic Microwave Phase Shifters. IEEE J. Quantum Electron..

[26] Chen L.R. (2017). Silicon Photonics for Microwave Photonics Applications. J. Light. Technol..

[27] Pan S., Yao J. (2017). Photonics-Based Broadband Microwave Measurement. J. Light. Technol..

[28] Zhu Y.L., Wu B.L., Li J., Wang M.G., Xiao S.Y., Yan F.P. (2022). Switchable instantaneous frequency measurement by optical power monitoring based on DP-QPSK modulator. Chin. Phys. B.

[29] Li S., Liu Y., Xing G., Ma X., Yu W., Zhang L., Luo Q., Liang X. UWB real-time spectrum measurement based on microwave photonics. Proceedings of the Seventeenth National Conference on Laser Technology and Optoelectronics.

[30] Shi N., Hao T., Li W., Li M. (2020). A Compact Multifrequency Measurement System Based on an Integrated Frequency-Scanning Generator. Appl. Sci..

[31] Zhu W., Li J., Yan M., Pei L., Ning T., Zheng J., Wang J. (2023). Photonic Multiple Microwave Frequency Measurement System with Single-Branch Detection Based on Polarization Interference. Electronics.

[32] Yang D., Zhang Y., Yang F., Yang M., Cao Y. (2024). Photonics-Based Multifunction System for Radar Signal Transmit-Receive Processing and Frequency Measurement. Micromachines.

[33] Lam D., Buckley B., Lonappan C., Madni A., Jalali B. (2015). Ultra-wideband instantaneous frequency estimation. IEEE Instrum. Meas. Mag..

[34] Bai Z., Li S., Xue X., Zheng X. (2023). Ultra-wideband instantaneous frequency measurement based on differential photonic time-stretch. Opt. Commun..

[35] Ji Q., Zhang J., Zhang J., Ma D. (2024). Broadband reconfigurable instantaneous microwave multifrequency measurement system based on a nonuniform optical frequency comb. Appl. Opt..

[36] Li S., Liu A., Ma X., Yu W., Liu Y., Li Y., Xing G. (2024). Broadband Instantaneous Frequency Measurement Using Frequency-to-Time Mapping and Channelization. Photonics.

[37] Zhang J., Ji Q., Zhang J. (2025). Wideband Reconfigurable Instantaneous Microwave Multi-frequency Measurement System Based on an Optical Frequency Shifter and Optical Frequency Comb. Curr. Opt. Photonics.

[38] Shi D., Li G., Jia Z., Wen J., Li M., Zhu N., Li W. (2021). Accuracy enhanced microwave frequency measurement based on the machine learning technique. Opt. Express.

[39] Nazemosadat E., García S., Gasulla I. Broadband Microwave Frequency Measurement Using a Heterogeneous Multicore Fiber. Proceedings of the 2022 IEEE International Topical Meeting on Microwave Photonics (MWP).

[40] Tao Y., Yang F., Tao Z., Chang L., Shu H., Jin M., Zhou Y., Ge Z., Wang X. (2022). Fully On-Chip Microwave Photonic Instantaneous Frequency Measurement System. Laser Photon. Rev..

[41] Cai J., Wen A., Li P., Zhuo H., Zhang W., Dong Y.Y. (2023). Instantaneous photonic frequency measurement based on compressive sensing. Opt. Commun..

[42] Dai W., Yang B., Yang S., Zhai Y., Ou J., Chi H. (2025). Photonic compressive sensing of microwave signals with enhanced compression ratio and frequency range via optical pulse random mixing. Opt. Lett..

[43] Jiang H., Marpaung D., Pagani M., Vu K., Choi D.-Y., Madden S.J., Yan L., Eggleton B.J. (2016). Wide-range, high-precision multiple microwave frequency measurement using a chip-based photonic Brillouin filter. Optica.

[44] Long X., Zou W., Chen J. (2017). Broadband instantaneous frequency measurement based on stimulated Brillouin scattering. Opt. Express.

[45] Hao T., Tang J., Li W., Zhu N., Li M. (2018). Microwave photonics frequency-to-time mapping based on a Fourier domain mode locked optoelectronic oscillator. Opt. Express.

[46] Hao T., Tang J., Shi N., Li W., Zhu N., Li M. (2019). Multiple-frequency measurement based on a Fourier domain mode-locked optoelectronic oscillator operating around oscillation threshold. Opt. Lett..

[47] Wu K., Li J., Zhang Y., Dong W., Zhang X., Chen W. (2015). Multiple microwave frequencies measurement based on stimulated Brillouin scattering with ultra-wide range. Optik.

[48] Jiao W., You K., Sun J. (2019). Multiple microwave frequency measurement with improved resolution based on stimulated Brillouin scattering and nonlinear fitting. IEEE Photonics J..

[49] Li X., Shi T., Ma D., Chen Y. (2024). Channelized Analog Microwave Short-Time Fourier Transform in the Optical Domain. IEEE Trans. Microw. Theory Tech..

[50] Jiang H., Marpaung D., Pagani M., Yan L., Eggleton B.J. Multiple Frequencies Microwave Measurement using a Tunable Brillouin RF Photonic Filter. Proceedings of the 2015 Conference on Lasers and Electro-Optics Pacific Rim.

[51] Zhu B., Tang J., Zhang W., Pan S., Yao J. (2021). Broadband instantaneous multi-frequency measurement based on a Fourier domain mode-locked laser. IEEE Trans. Microw. Theory Tech..

[52] Zhou F., Chen H., Wang X., Zhou L., Dong J., Zhang X. (2018). Photonic Multiple Microwave Frequency Measurement Based on Frequency-To-Time Mapping. IEEE Photonics J..

[53] Zhao M., Wang W., Shi L., Che C., Dong J. (2023). Photonic-Assisted Microwave Frequency Measurement Using High Q-Factor Microdisk with High Accuracy. Photonics.

[54] Singh K., Preußler S., Misra A., Zhou L., Schneider T. (2022). Photonic Microwave Frequency Measurement with High Accuracy and Sub-MHz Resolution. J. Light. Technol..

[55] Zhang W., Yao J. A silicon photonic integrated frequency-tunable microwave photonic bandpass filter. Proceedings of the International Topical Meeting on Microwave Photonics (MWP).

[56] Marpaung D., Morrison B., Pagani M., Pant R., Choi D.-Y., Luther-Davies B., Madden S.J., Eggleton B.J. (2015). Low-power, chip-based stimulated Brillouin scattering microwave photonic filter with ultrahigh selectivity. Optica.

[57] Liu Y., Yu Y., Yuan S., Xu X., Zhang X. (2016). Tunable megahertz bandwidth microwave photonic notch filter based on a silica microsphere cavity. Opt. Lett..

[58] Liu L., Yang Y., Li Z., Jin X., Mo W., Liu X. (2017). Low power consumption and continuously tunable all-optical microwave filter based on an opto-mechanical microring resonator. Opt. Express.

[59] Liu X., Sun C., Xiong B., Wang J., Wang L., Han Y., Hao Z., Li H., Luo Y., Yan J. (2016). Broadband tunable microwave photonic phase shifter with low RF power variation in a high-Q AlN microring. Opt. Lett..

[60] Burla M., Crockett B., Chrostowski L., Azana J. Ultra-high Q multimode waveguide ring resonators for microwave photonics signal processing. Proceedings of the 2015 International Topical Meeting on Microwave Photonics (MWP).

[61] Qiu H., Zhou F., Qie J., Yao Y., Hu X., Zhang Y., Xiao X., Yu Y., Dong J., Zhang X. (2018). A Continuously Tunable Sub-Gigahertz Microwave Photonic Bandpass Filter Based on an Ultra-High-Q Silicon Microring Resonator. J. Light. Technol..

[62] Burla M., Wang X., Li M., Chrostowski L., Azanã J. (2016). Wideband dynamic microwave frequency identification system using a low-power ultracompact silicon photonic chip. Nat. Commun..

[63] Zhu B., Zhang W., Pan S., Yao J. (2019). High-sensitivity instantaneous microwave frequency measurement based on a silicon photonic integrated fano resonator. J. Light. Technol..

[64] Chen Y., Zhang W., Liu J., Yao J. (2019). On-chip two-step microwave frequency measurement with high accuracy and ultra-wide bandwidth using add-drop micro-disk resonators. Opt. Lett..

[65] Wang X.U., Zhou F., Gao D., Wei Y., Xiao X.I., Yu S., Dong J., Zhang X. (2019). Wideband adaptive microwave frequency identification using an integrated silicon photonic scanning filter. Photonics Res..

[66] Cao R., He Y., Zheng R., He Z., Zhi Y., Wang X., Zhang J., Yao J., Yao J. (2022). Microwave frequency measurement using a silicon integrated microring resonator. Appl. Opt..

[67] Hong X., Liu H.H., Wang B., Zhang W. Breaking the Resolution Limitation in Fast Sweeping Photonic-Assisted Microwave Frequency Identification. Proceedings of the 2024 International Topical Meeting on Microwave Photonics (MWP).

[68] Liu L., Jiang F., Yan S., Min S., He M., Gao D., Dong J. (2015). Photonic measurement of microwave frequency using a silicon microdisk resonator. Opt. Commun..

[69] Zhou P., Tang Z., Zhu J., Li N. (2023). Instantaneous Frequency Measurement Using Photonics-Assisted Broadband Signal Generation and Processing. IEEE Microw. Wirel. Technol. Lett..

[70] Shi J., Zhang F., Ye X., Yang Y., Ben D., Pan S. (2019). Photonics-based dual-functional system for simultaneous high-resolution radar imaging and fast frequency measurement. Opt. Lett..

[71] Shi J., Zhang F., Ben D., Pan S. (2020). Simultaneous Radar Detection and Frequency Measurement by Broadband Microwave Photonic Processing. J. Light. Technol..

[72] Zhou Y., Zhang F., Shi J., Pan S. (2020). Deep neural network-assisted high-accuracy microwave instantaneous frequency measurement with a photonic scanning receiver. Opt. Lett..

[73] Shi J., Zhang F., Zhou Y., Pan S., Wang Y., Ben D. (2020). Photonic scanning receiver for wide-range microwave frequency measurement by photonic frequency octupling and in-phase and quadrature mixing. Opt. Lett..

[74] Wang G., Meng Q., Li Y.J., Li X., Zhou Y., Zhu Z., Gao C., Li H., Zhao S. (2023). Photonic-assisted multiple microwave frequency measurement with improved robustness. Opt. Lett..

[75] Chen X., Dong W., Kong Z., Li G., Wang L., Chen S., Li M., Zhu N., Li W. (2023). Precise Multiple Frequency Identification Based on Frequency-to-Time Mapping and Cross-Correlation. J. Light. Technol..

[76] Xie X., Luo C., Tang H., Du J., Li M., Li W. (2024). Photonic-Assisted Multi-Tone Microwave Frequency Measurement Based on Pulse Identification. Photonics.

[77] Yang Z., Liu Z., Jiang Y., Liu H., Li J., Dong W. (2024). High-Precision Photonics-Assisted Two-Step Microwave Frequency Measurement Combining Time and Power Mapping Method. Sensors.

[78] Nguyen T.A., Chan E.H.W., Minasian R.A. (2014). Instantaneous high-resolution multiple-frequency measurement system based on frequency-to-time mapping technique. Opt. Lett..

[79] Huang Y., Guo J., Jiang L., Huang Y., Lou Y., Li X., Chen Y., Yan L. (2023). Photonics-assisted two-step microwave frequency measurement based on frequency-to-time mapping. Opt. Quantum Electron..

[80] Minasian R.A., Yi X. Stimulated Brillouin scattering based microwave photonic signal processors. Proceedings of the International Conference on Transparent Optical Networks.

[81] Wei Y., Wang X., Miao Y., Chen J., Wang X., Gong C. (2022). Measurement and analysis of instantaneous microwave frequency based on an optical frequency comb. Appl. Opt..

[82] Shen J., Wu S., Li D., Liu J. (2019). Microwave multi-frequency measurement based on an optical frequency comb and a photonic channelized receiver. Appl. Opt..

[83] Zhu W., Li J., Yan M., Pei L., Ning T., Zheng J., Wang J. (2022). Multiple microwave frequency measurement system based on a sawtooth-wave-modulated non-flat optical frequency comb. Appl. Opt..

[84] Shen Z., Jin C., He Q., Zhang Z., Zhao Y. (2019). Photonics-Assisted Non-Scanning High-Accuracy Frequency Measurement Using Low-Speed Components. IEEE Photonics J..

[85] Lu X., Pan W., Zou X., Bai W., Li P., Yan L., Teng C. (2020). Wideband and Ambiguous-Free RF Channelizer Assisted Jointly by Spacing and Profile of Optical Frequency Comb. IEEE Photonics J..

[86] Huang C., Chan E.H.W., Hao P., Wang X. (2023). Wideband High-Speed and High-Accuracy Instantaneous Frequency Measurement System. IEEE Photonics J..

[87] Zhao Z., Zhu K., Lu L., Lu C. (2020). Instantaneous microwave frequency measurement using few-mode fiber-based microwave photonic filters. Opt. Express.

[88] Liu L., Xue W. (2020). Instantaneous Microwave Frequency Measurement Based on Two Cascaded Photonic Crystal Nanocavities. IEEE Photonics J..

[89] Li J., Pei L., Ning T., Zheng J., Li Y., He R. (2020). Measurement of Instantaneous Microwave Frequency by Optical Power Monitoring Based on Polarization Interference. J. Light. Technol..

[90] Li Y., Kuse N., Fermann M.E. Photonic-assisted Wideband Microwave Measurement. Proceedings of the IEEE MTT-S International Microwave Symposium Digest.

[91] Yao Y., Zhao Y., Wei Y., Zhou F., Chen D., Zhang Y., Xiao X., Li M., Dong J., Yu S. (2022). Highly Integrated Dual-Modality Microwave Frequency Identification System. Laser Photon. Rev..

[92] Ding J., Zhu D., Yang Y., Ni B., Zhang C., Pan S. (2023). Simultaneous Angle-of-Arrival and Frequency Measurement System Based on Microwave Photonics. J. Light. Technol..

[93] Li Z., Zhang X., Chi H., Zheng S., Jin X., Yao J. (2012). A reconfigurable microwave photonic channelized receiver based on dense wavelength division multiplexing using an optical comb. Opt. Commun..

[94] Jiang Y., Li J., Yan M., Tian C., Pei L., Ning T. (2024). Channelized multi-frequency measurement system based on asymmetric double sideband detection. Appl. Opt..

[95] Nguyen L.V.T. (2009). Microwave photonic technique for frequency measurement of simultaneous signals. IEEE Photonics Technol. Lett..

[96] Ye C., Fu H., Zhu K., He S. (2012). All-optical approach to microwave frequency measurement with large spectral range and high accuracy. IEEE Photonics Technol. Lett..

[97] Pan S., Zhang F., Zhou Y. (2021). Instantaneous frequency analysis of broadband LFM signals by photonics-assisted equivalent frequency sampling. Chin. Opt. Lett..

[98] Wen H., Zheng H., Mo Q., Velázquez-Benítez A.M., Xia C., Huang B., Liu H., Yu H., Sillard P., Lopez J.E.A. (2017). Few-mode fibre-optic microwave photonic links. Light. Sci. Appl..

[99] Nickel D.V., Villarruel C., Koo K., Bucholtz F., Haas B. (2017). Few Mode Fiber-Based Microwave Photonic Finite Impulse Response Filters. J. Light. Technol..

[100] Nickel D.V., Maize I.M. (2022). Investigation of a balanced microwave photonic link utilizing a single few-mode fiber. Appl. Opt..

[101] Zhao J., Zhang H., Yang Z., Xu J., Xu T., Wang C. (2020). Few-Mode Fibers with Uniform Differential Mode Group Delay for Microwave Photonic Signal Processing. IEEE Access.

[102] Nazemosadat E., Gasulla I. (2020). Dispersion-tailored few-mode fiber design for tunable microwave photonic signal processing. Opt. Express.

[103] Nazemosadat E., Gasulla I. (2022). Reconfigurable Few-Mode Fiber-Based Microwave Photonic Filter. J. Light. Technol..

[104] Nickel D.V., Maize I.M., Singley J.M. (2023). One kilometer balanced analog photonic link based on a single multicore fiber. Opt. Contin..

[105] Liu S., Chen D., Cui T., Zhang Y. Microwave photonics instantaneous frequency measurement scheme assisted by machine learning method. Proceedings of the Fourth Optics Frontier Conference (OFS 2024).

[106] Jia Q., Li J., Wei C., Liu J. (2023). Microwave Photonic Reconfigurable High Precision Instantaneous Frequency Measurement System Assisted by Stacking Ensemble Learning Method. J. Light. Technol..

[107] Jia Q., Xiang Z., Li D., Liu J., Li J. (2024). Machine-Learning-Assisted Instantaneous Frequency Measurement Method Based on Thin-Film Lithium Niobate on an Insulator Phase Modulator for Radar Detection. Sensors.

[108] Chen D., Liu S., Cui T., Cai C., Zhang Y., Zhou B., Yang X., Liu X. (2025). Instantaneous frequency measurement scheme based on scalable structure and machine learning assistance. Opt. Fiber Technol..

[109] Polikar R., Zhang C., Ma Y. (2012). Ensemble Learning. Ensemble Machine Learning.

[110] Zou W., Chen J., Zou X., Xu S., Li S. (2019). Optimization of the Brillouin instantaneous frequency measurement using convolutional neural networks. Opt. Lett..

[111] Zhang W., Yao J. (2020). Photonic integrated field-programmable disk array signal processor. Nat. Commun..

[112] Zhang W., Wang B. (2023). On-Chip Reconfigurable Microwave Photonic Processor. Chin. J. Electron..

